# Indents

**Published:** 1914-06-15

**Authors:** 


### INDENTS.

☞Brief Articles of Technical or Scientific Value will receive notice and comment. Contributions invited.

*Saliva Tubes.* To meet the objection of unclean saliva tubes, a Frenchman has made a celluloid tube that is sterilized when made and sold in a sealed envelope. It is so inexpensive that it can be used once and thrown away. In this way much of the serious objection to the ordinary saliva tubes can be met both to the comfort of the dentist and patient.

*Radium Mouth Wash for Pyorrhoea.* Leger-Dorez is experimenting with a mouth wash of radium sulphate in a water solution for the treatment of inflammatory peridentitis with satisfactory results. He uses one-half miligram of radium sulphate mixed with five grains of barium sulphate in five quarts of water. The mouth is washed thoroughly for fifteen minutes daily with the barium water, which will cause a resolution of the inflammatory condition in from four to six days. It must be a rather expensive treatment. Similar results can be secured with much simpler remedies. Applications of iodine and aconite with a mouth wash of tincture of myrrh or calendula will do the same.

*Baldness from Affected Teeth.* Drs. Ferrier and Schmeltz report in *Sud. Est Dentoire* January 1, 1914, a case where a patient suffered from headache and loss of hair in the regions of the right temple, the left parietal region and the left occipital region. These regions were painful to the touch especially when the hair was combed. A number of roots of teeth were extracted and such teeth as required it were filled. In one months time the hair began to grow in again in all the affected areas.

There was no toothache at any time, but the alopecia was apparently caused by the alveolar irritation about the diseased roots.

*Too Many Patients.* Most dentists have more patients than they can properly serve in the time they ought to work. What most of us need is fewer patients whom we will serve adequately and get better compensation, and have more time for out of doors and fresh air, so that we shall be physically fit to earn the fees we get.—*Bush.*

*Selling Service rather than Fillings.* One of the undignified habits of the dental profession is that of charging for fillings; cement, amalgam or gold, or for plates, or crown or bridges, etc. Who ever heard of a physician charging one price for uncoated pills and another for his sugar coated remedies? We should stop selling or talking about the kind of filling, but talk about the quality or adaptibility of the service you render. The patient wants an adequate service and he is willing to trust your judgment if he has received adequate and satisfactory service at your hands before. The key note of success is to render proper and satisfactory service all the time. This is the true professional ideal and it applies to every operation of any kind that the dentist is called upon to make.—Dr. Bush in *Dominion Dental Journal.*

*Bad Teeth.* Of 121,369 physical defects noted in the school children of Boston, 108,984 were bad teeth.

*Broaching the Pulp.* If you have trouble in snaring the pulp with your barbed broach, put two broaches in your holder and a slight twist in the canal will engage the most elusive pulp.—F. H. Wilkinson in *Digest.*

*Tempering Glasses.* When your tumblers are new put them in a pot of cold salt water and slowly bring it to the boiling point and allow the water to cool slowly. The glassware will not be so easily broken and be more durable.—Keith Sutherland, Sydney, Australia in *Digest.*

*Splicing Rubber Tubing.* To join two rubber tubes of different sizes, slip the smaller one into the large one, dampen with collodion, wrap a wisp of cotton saturated with collodion around the joint. The two can be joined in this way, gas and air-tight, and the joint can be smoothed up to present a perfectly workman-like appearance.—J. Scott Walker in *Western Journal.*

*The Dental Nurse, The Dental Assistant and the Dental Mechanic.* In some parts of the United States there is an effort among dentists to have organized a regular dental nursing profession which shall have Legislative recognition under a state license. In reality it would be a partial dental license. For some years dentists have been training their assistants to perform prophylaxis and it is out of this has grown the idea of State recognition. The idea has gone far enough to have a school organized for training nurses.

The dental assistant, as understood in Canada and most other countries, assists the dentist in operations, both at the chair and in the laboratory, besides doing many other things which are necessary to save the dentist's time. In no case does the assistant perform dental operations for patients.

In some of the Provinces of Canada there is recognized by the dental Act an assistant who is a graduate in dentistry but has no license in the Province in which he is practising. This is simply a means of covering as recognized in Great Britain. Such an assistant may legally perform any dental operation so long as he receives his remuneration from a licensed dentist and not directly from the patient. A sort of bond servant. He has the necessary skill to perform dental operations but no legal right to collect the fee. Such a legalized assistant cannot be justified before the people. If he has the necessary skill to perform dental operations he should have a license.

The dental mechanic is expected to do all his operations in the laboratory and in no case see patients. In most cases the mechanic can do the dental mechanical work for several dentists.

Now why all these forms of help for the dentist? There is dental surgery and dental mechanics. The scope of each is fairly well defined, but the success or failure of the one largely depends upon the other. The whole difficulty is that the knowledge and skill to become a dental surgeon is so wide and the consequence of his operations so important to the health of the patient that a fee must be charged that is beyond the reach of many, while on the other hand many dental operations are merely mechanical and do not require either knowledge or skill beyond that of a mechanic. It is in the interest of the public from an economic standpoint that every person shall perform the highest service that he is capable of. It is waste of money to educate a dental surgeon and have him spend his time performing operations that might be done by a nurse, an assistant or dental mechanic.

In all this demand for dental assistants there is something of the captain of industry, with the added legalizing of the captain's position. If only a dentist with a license can have all kinds of assistants he has a monopoly which was never intended. The only logical position is for the dentist to perform his own dental operations and employ what assistants he can use, but none of them who has not a license, should be allowed to see patients.—Dr. A. E. Webster in *Dominion Dental Journal.*

*Filling Root Canals.* A canal plugger should be used to secure the approximate size of the foramen and a gutta percha cone selected, the diameter of which is only slightly larger. At the time of measuring the foramen we can also note the length of the canal. It is a mistake to use a long cone, for it can not be properly handled, and it is impossible to pack such a cone and fully seal the cavity. The cone should not be longer than three millimeters. The canal plugger should be heated and the cone attached to the plugger and placed on the tray ready for immediate use. Eucalyptol should now be carried on a broach wound with a bit of cotton to moisten the entire length of the lingual canal, and for this purpose it is important that the broach be of proper size so that it will not go beyond the apex and injure the peridental membrane. The slightest injury to the apical membrane might produce a hemorrhage which would result in an imperfect sealing of the foramen. The tooth should be slightly warmed by the hot air syringe and the gutta percha point dipped in eucalyptol and immediatly carried to the apex. While the indication of slight pain from the patient may mean that the cone has reached the apex, that sign is by no means infallible, for air forced ahead of the cone may account for this sensation of pain. The use of eucalyptol is indicated for several reasons:

First—It will displace moisture, because eucalyptol has a greater affinity for dentin.

Second—It is a slight solvent for gutta percha and causes the gutta percha to adhere to the wall.

Third—It is a lubricant, making it easier to force the gutta percha into the small canals; and

Fourth—It is antiseptic.

The filling of the root to the pulp chamber should continue in the same manner described, except that the cones should become relatively larger in diameter due to the enlarging of canal, and the tooth should be warmed several times to insure the thorough packing of the gutta percha. If the root canal plugger is sufficiently large so that the gutta percha will be forced ahead of the point instead of puncturing it a great amount of pressure can be used during the entire process of packing, resulting in a dense filling that will hermetically seal the canal.

The only variation in the filling of the mesio-buccal and disto-buccal canals is that I would usually use chloro-percha to help in the filling of these smaller canals. But this is easier said than done. In these smaller canals I am satisfied that many times we fail because our pluggers and broaches are not delicate enough. Another common cause of failure is the too generous use of chloro-percha or using chloro-percha of improper consistency. The smallest amount of cotton wound tightly on a broach with a small amount of chloro-percha carried to the apex or as close as possible should be followed immediately by the cone that has been previously prepared and this should be pressed, if possible, to the apex. Much of this must be done very quickly and it is essential there be no failures in any part of the operation.—Dr. Gethrow in *Review*.

*Soldering Backings to Platinum Pin Teeth.* Much trouble is sometimes caused through carelessness when backing teeth with gold or other metal. To avoid it after the case has been waxed-up, invested, and scalded out, it should be placed on an asbestos or fire-clay slab and slightly heated until all the moisture has been driven off. The blow-pipe flame should then be applied to the case and finally directed upon the solder.

If the case is allowed to remain upon the Bunsen, beyond the drying-off stage, rapid and excessive oxidation takes place, which is likely to be absorbed by the platinum pins, and to render them brittle and very liable to fracture.—*Ash's Monthly.*

*A New Physiological Haemostatic.* Attention is drawn in a recent number of *Il Morgagni* (No. 17, March 22, 1914, Milano) to the use of the fibrin ferment of the blood as a valuable haemostatic. This substance is to be found in all the cells of the organism and extracts of all fresh organs contain it. As prepared by

Zanoni it exists as a fine powder, of a colour varying from light to dark yellow, slightly unctuous to the touch, and with an odour like that of goose fat. It is slightly hygroscopic, and but little changed by light and air, sparingly soluble in water and chloroform, and less still in 95 per cent. alcohol. Aqueous solutions are not limpid, are markedly opalescent, and leave an insoluble residue; they cannot be filtered, since most of the ferment remains on the filter. They must therefore be used as they are. This substance, which Zanoni has named "coaguline," reduces the time required for the formation of blood clots from fifteen minutes to one minute. Coaguline promises to be of use in checking superficial haemorrhages and in bleeding from the nose and mouth. To ensure a full haemostatic action it is not enough to apply the powder or solution to the bleeding surface since the blood would wash it away before it had time to complete its coagulating action; it must be kept *in situ* for a short time with a pledget of cotton or gauze and with slight pressure.

*Disinfection.* Japanese paper blocks should be fixed to head-rests; that tumblers, after use, should be left for fully five minutes in boiling water; that both before and after each operation the hands, after thorough cleansing and sterilizing in 70 per cent. alcohol, must be dried on a clean fresh towel; that clean white coats should be used at the rate of at least one per day; that while filling and burnishing instruments require but little attention, excavators should be first mechanically cleaned and then passed through the flame of spirit lamp or Bunsen burner before they can safely be used. Mirrors, drills, stones, clamps, tweezers, nerve instruments, etc., demand special care. Three methods of dealing with them are available—they may be sterilized by immersion in an antiseptic solution; in steam or in boiling water; in formalin vapour. The same objection applies to the use of dry heat. Preference must be given to steam or boiling water as being at once thorough and quick, five minutes' immersion being fully sufficient. Rusting may be avoided by the addition of a little soda to the water. Certain instruments, such as hand-pieces, elbow-joints, etc., which cannot be laid in water, may, with the help of the vulcanizer, be cleaned by steam under pressure; or there are apparatus to be had fitted with oil or thymol sterilizing baths. Formalin vapor is unsatisfactory, unless accompanied by steam. Needles used in exploring nerve-canals should not be used a second time. Probes and Beutelrock drills must be cleansed with the utmost care.—D. Fleischman in *American Dental Journal.*

*Pyorrhea Alevolaris Treatment.* In the treatment of pyorrhea alveolaris the author has adopted the following method:

1. Urine-analysis. Splinting of all loose and slightly loose teeth by means of silk ligatures. The mouth is then copiously irrigated with an antiseptic solution and the pockets are packed with gauze impregnated in a 25 per cent. solution of phenosulfonic acid. The patient is then dismissed for twenty-four hours when the teeth and roots are thoroughly scaled, however, without removing the ligatures.

2. At the following sitting the silk ligatures are replaced by gold ones and after the teeth are again solidly splinted the gingival pockets are treated each with two centigrams of a preparation consisting of

| | |
|---|---|
| Metallic iodin | 0.50 gms. |
| Carbolic acid | 2.00 gms. |
| Tincture of aconite | 6.00 gms. |
| Glycerine | 100.00 gms. |

After the solution has been injected into the pockets these are again packed with carbolized gauze and the patient is instructed to use several times daily a mouth wash of sodium perborate 3:100. This treatment is given at intervals of three days and during a period of from 5 to 6 weeks.—Dr. F. E. Hart in *Revista Dental.*

*Fees.* There is no conflict between ethics and business and if there appears to be at any time, the fault lies with what we are pleased to call business.

Business to be worthy to get such universal attention from mankind must be founded upon honor and in truth. Ethics has no better pedigree, in fact, the same. It is a part of a man's obligations to have enough business sense about him that he may not eventually become a charge upon the public nor yet those for whom he is responsible. This ideal can be achieved by the proper attention to business, and in doing this one does not need to sell his soul to Mammon nor make wealth his ideal. He need not devote his time to this end to the exclusion of all finer pursuits and sentiments to be found in a well ordered existence upon earth. He is as much a fool to be sordid as he is to be profligate. There is a proper and well balanced career open to every normal man and it is his duty to stay within the lines of sense upon either side. Large savings are not necessary to gain safety, but rather continuous savings and a reasonable care of your accumulations. Large dividends are not necessary but rather sure ones. It avails us not to merely save money by economy. We must keep our savings. This is as reasonably certain of accomplishment as any other human ambition if we do not fall victim to greedy desires. Nor is it necessary to gain large fees in order to lay by money. It can be done on any fee short of the absolutely negative compensation. There is a very fixed relationship between the fee a man gets and social expense necessary to get it. There is a Polo club fee and a Church social fee and the latter will always win in the end. It may not be as spectacular as the first, but it is sounder. Next to women, the fee question has caused more bickering argument and controversy than anything else in the world. Societes have been rent asunder upon the subject, and learned scientific gentlemen have forgotten their formulas and tomes, flayed the air with wild gesticulations and thundered forth oratory in their patriotic effort to "raise" fees. Dental journals have and are devoting page upon page and wearing out tons of type in frantic endeavor to prove that we should get better fees. All this is vain, futile, stale and unprofitable. It is worse than useless, it is belittling, it verges on the unethical and is sacrificing that finer professional dignity which it should be the duty of every loyal man to uphold and defend.

Fees are about as fixed a quantity as a broken broach in a root canal. You cannot change them much. The fee is the attribute of the clientele and not the dentist to any great extent. If you go to a small country community and have sense enough to make yourself a valuable and respected part of that people, a little observation upon its habits of living, the kind of dress, the kind of supplies and social character of the citizens will determine your just and equitable fee. If you do not like it, move on, but do not rail at the profession. If you locate in the fashionable suburb of some rich city, another fee is plainly indicated. It is right for you to get it, if you can. The service should be of the same standard of integrity in either case, but not at all of the same elaboration. If you are an overall man in a Tuxedo community, you will fail; if you are a Tuxedo man in an overall community, it is your own fault. You are lacking in true business sense in either case. In large communities where many kinds of clientele dwell together, many men of our calling may work side by side and each will receive his appropriate reward.—Dr. C. C. Allen in *Western Journal.*

*Diseased Teeth a cause of Tuberculosis.* Let the fact stand out, as prominently as possibly it can, that no other factor plays a more important role in the incidence of tuberculosis than teeth with carious cavities, mortified and infected pulps, abscesses upon their roots and all the sequelae of these pathologic conditions. It is no longer a case of stating in a cursory way that diseased teeth or gums may be the starting point of a bacillosis, but that they are in by far the largest number of instances the primal etiologic factor of localized and generalized tuberculosis. It is from a sense of duty to the cause to which they are dedicated that we venture to call the attention of the medical and dental professions to these oft-neglected channels of tubercular infection. The dental profession alone can accomplish much toward the eradication of the white plague, by continuous hammering upon these facts from the standpoint of the man who "knows" and not merely "thinks."

If the dental profession will only avail itself of the resources at its command and fight the battle for better bodies because of better teeth with all the weapons in its own armamentarium—the fight for better teeth not alone for the sake of sound masticatory organs, but for the sake of increasing the sum total of human efficiency it will not be long before its salutary influence will reach every corner of the globe and the riches it will reap in the shape of lives saved or made more useful will more than compensate its workers for the continued expenditure of mental and physical energy implied by the nature of the aims to be attended.—Julio Endelman in *Pacific Gazette.*

*Value of Buccal Flanges.* As an illustration of the value of the widening of the gum; alongside the posterior teeth in the multitude of cases of flat narrow jaws, is the following:

A patient who had worn a denture for a year was often calling for help, or relief from irritated gums, owing largely to the movability of the plate, disappeared for a year by absence from town. She put it in appearance saying, "If I had to endure this another year I would jump into the lake."

I took the plate and placed wax flanges on it and asked her to call the next day. She did so and said it was an improvement. I replaced the wax with rubber and she called a week later and smilingly said, "Well I am not going to jump into the lake."

In my own case, I have this experience, while one would suppose that berries and crust crumbs would be constantly getting under such movable plates, I rarely ever have any trouble. It is the cheeks that help hold the plate, as they lay on these flanges.—Dr. L. P. Haskell in *Dental Review.*

